# Food Insecurity Among Older Urban Subsidized Housing Residents: The Importance of Social Connectedness

**DOI:** 10.1093/geroni/igab046.524

**Published:** 2021-12-17

**Authors:** Judith Gonyea, Arden O'Donnell, Alexaandra Curley

**Affiliations:** 1 Boston University, Boston, Massachusetts, United States; 2 Case Western Reserve University, Cleveland, Ohio, United States

## Abstract

Poverty and food insecurity are associated with poor health in later life. Although housing is recognized as a social determinant of health; relatively little research has explored food insecurity in the marginalized population of older subsidized housing residents. In this study, we examined factors associated with food insecurity and particularly how social connectedness was associated with food insecurity. We hypothesized that social connection measures (i.e., loneliness, sense of belonging) independent of sociodemographic, health and food program variables would contribute to food insecurity. Our data are from interviews with 216 residents ages 55-plus (50% Black, 45% LatinX). The 6-item USDA Household Food Security Survey found high rates of food insecurity, 40% for ages 55-69 and 20% for ages 70-plus. Multivariate logistic regression models revealed that loneliness was significantly related to food insecurity even after other factors were controlled. Discussion centers on strategies for addressing social risk factors to ameliorate food insecurity.

